# 2-Chloro-1-[4-(2-fluoro­benz­yl)piperazin-1-yl]ethanone

**DOI:** 10.1107/S1600536811006180

**Published:** 2011-02-26

**Authors:** Cunlong Zhang, Xin Zhai, Furen Wan, Ping Gong, Yuyang Jiang

**Affiliations:** a Key Laboratory of Original New Drug Design and Discovery of the Ministry of Education, Shenyang Pharmaceutical University, Shenyang, Liaoning 110016, People’s Republic of China; b Deparment of Chemistry, Northeast Normal University, Changchun 130024, People’s Republic of China; c The Key Laboratory of Chemical Biology, Guangdong Province, Graduate School at Shenzhen, Tsinghua University, Shenzhen 518055, People’s Republic of China; d School of Medicine, Tsinghua University, Beijing 100084, People’s Republic of China

## Abstract

In the title compound, C_13_H_16_ClFN_2_O, the piperazine ring is flanked by 1-(2-fluoro­benz­yl)piperazine and adopts a chair conformation. The dihedral angle between the fluoro­phenyl ring and the four planar C atoms (r.m.s. = 0.0055 Å) of the piperazine chair is 78.27 (7)°, whereas the dihedral angle between the four planar C atoms of the piperazine chair and the ethanone plane is 55.21 (9) Å; the Cl atom displaced by1.589 (2) Å out of the plane.

## Related literature

For the synthesis of related compounds, see: Contreras *et al.* (2001 ▶); Capuano *et al.* (2002 ▶). For their use as inter­mediates in the synthesis of anti-inflammatory agents or CCR1 antagonists, see: Rolland & Duhault (1989 ▶); Kaufmann (2005 ▶); Tanikawa *et al.* (1995 ▶); Xie *et al.* (2007 ▶).
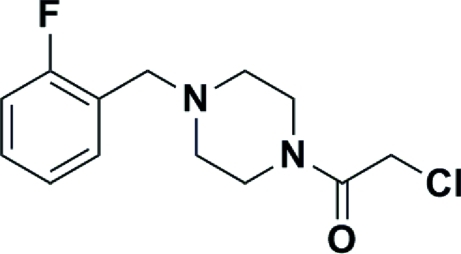


## Experimental

### 

#### Crystal data

C_13_H_16_ClFN_2_O
*M* *_r_* = 270.73
Orthorhombic, 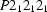

*a* = 7.9350 (5) Å
*b* = 8.4610 (4) Å
*c* = 19.0040 (11) Å
*V* = 1275.89 (12) Å^3^
*Z* = 4
Mo *K*α radiation
μ = 0.30 mm^−1^
*T* = 291 K
0.30 × 0.30 × 0.20 mm

#### Data collection

Bruker APEXII CCD diffractometer
Absorption correction: multi-scan (*SADABS*; Bruker, 2001 ▶) *T*_min_ = 0.566, *T*_max_ = 0.716
12886 measured reflections
3001 independent reflections
2550 reflections with *I* > 2σ(*I*)
*R*_int_ = 0.033

#### Refinement

*R*[*F* ^2^ > 2σ(*F* ^2^)] = 0.035
*wR*(*F* ^2^) = 0.085
*S* = 1.01
3001 reflections
165 parameters
H-atom parameters constrained
Δρ_max_ = 0.33 e Å^−3^
Δρ_min_ = −0.18 e Å^−3^
Absolute structure: Flack (1983 ▶), 1255 Friedel pairs
Flack parameter: 0.03 (7)

### 

Data collection: *APEX2* (Bruker, 2003 ▶); cell refinement: *SAINT* (Bruker, 2001 ▶); data reduction: *SAINT*; program(s) used to solve structure: *SHELXS97* (Sheldrick, 2008 ▶); program(s) used to refine structure: *SHELXL97* (Sheldrick, 2008 ▶); molecular graphics: *DIAMOND* (Brandenburg, 1999) ▶; software used to prepare material for publication: *SHELXL97*.

## Supplementary Material

Crystal structure: contains datablocks I, global. DOI: 10.1107/S1600536811006180/si2316sup1.cif

Structure factors: contains datablocks I. DOI: 10.1107/S1600536811006180/si2316Isup2.hkl

Additional supplementary materials:  crystallographic information; 3D view; checkCIF report

## Figures and Tables

**Fig. 1.**
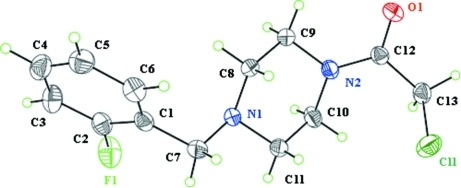
Molecular structure of the title compound showing displacement ellipsoids at the 30% probability level.

**Fig. 2.**
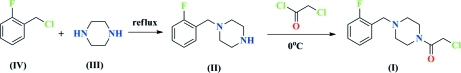
Synthesis of the title compound

## supplementary crystallographic information

### Comment

Piperazine derivatives similar to the title compound are well known as being useful for a variety of pharmaceutical indication, particularly as cardiotonic, neurotropic or anti-inflammatory agents (Kaufmann, 2005). The synthesis of related pyridazine compounds and their medicinal and pharmaceutical activity were reported (Contreras *et al.*, 2001; Capuano *et al.*, 2002). The use of related compounds as intermediates in the synthesis of antiinflammatory agents or CCRI antagonists can be studied in various patents (Rolland & Duhault, 1989; Kaufmann, 2005; Tanikawa *et al.*, 1995) and medicinal journal (Xie *et al.*, 2007). Moreover, we recently identified a series of compounds bearing various substituted benzylpiperazine moiety with potent antitumor activity by virtual screening approach (paper was being revised).

Herein, we report the synthesis of the title compound as one important representative of piperazine derivatives and its X-ray crystal structure. The molecule of (I) is shown in Fig. 1. The bond lengths and angles are within normal ranges. The piperazine ring in the molecule adopts a chair conformation. The dihedral angle between the fluorophenyl ring and the four planar C atoms (r.m.s. = 0.0055 Å) of the piperazine chair is 78.27 (7)°. Whereas the dihedral angle between the four planar C atoms of the piperazine chair and the ethanone plane is 55.21 (9)Å with the Cl atom about 1.589 (2) Å out of plane. In the crystal, there are no strong intermolecular hydrogen bonds to link the molecules.

### Experimental

All chemicals and solvents were obtained from commercial supplies and used without purification. To a solution of chloroacetic chloride (0.58 ml, 7.15 mmol) in CH_2_Cl_2_ (10 ml) was added, at 0 °C, 1-(2-fluorobenzyl)piperazine(II) (1.15 g, 5.90 mmol) dissolved in CH_2_Cl_2_ (20 ml) which was prepared from the reaction of anhydrous piperazine(III) and 1-(chloromethyl)-2-fluorobenzene(IV). The reaction mixture was stirred at room temperature for about 30 min until no 1-(2-fluorobenzyl)piperazine remained, as monitored by TLC. The mixture was poured into cold H_2_O (50 ml) and rendered alkaline with a 10% NaHCO_3_ aqueous solution and separated. The organic layer, dried over Na_2_SO_4_, was evaporated under reduced pressure to give 1.44 g of pure title compound as a yellow oil. Yield 90%; ^1^H NMR (400 MHz, CDCl_3_) δ 7.35 (dd, *J* = 7.4, 1.4 Hz, 1H), δ 7.23–7.29 (m, 1H), δ 7.12 (t, *J* = 7.2 Hz, 1H), δ 7.04 (t, *J* = 9.2 Hz, 1H), δ 4.05 (s, 2H), δ 3.64 (d, *J* = 5.2 Hz, 2H), δ 3.62 (d, *J* = 4.4 Hz, 2H), 3.52 (t, *J* = 5.0 Hz, 2H), δ 2.51 (dt, *J* = 15.6, 4.8 Hz, 4H); ^13^C NMR (100.6 MHz, CDCl_3_) δ 161.16, 131.24, 128.88, 123.88, 123.75, 115.27, 115.05, 54.87, 52.41, 52.00, 46.06, 41.93, 40.67.

### Refinement

All H atoms were positioned geometrically and refined using a riding model approximation with distances C—H = 0.93 Å for the benzene ring and 0.97 Å for C_sp3_ carbon atoms and *U*_iso_(H) = 1.2 times *U*_eq_(C). The absolute structure was determined by using the Flack parameter refinement with the TWIN/BASF instruction, and the coordinates of all atoms were inverted by instruction MOVE 1 1 1 - 1 in the final refinement with *SHELXL97*.

### Crystal data

**Table:**

C_13_H_16_ClFN_2_O	*F*(000) = 568
*M**_r_* = 270.73	*D*_x_ = 1.409 Mg m^−^^3^
Orthorhombic, *P*2_1_2_1_2_1_	Mo *K*α radiation, λ = 0.71069 Å
Hall symbol: P 2ac 2ab	Cell parameters from 25 reflections
*a* = 7.9350 (5) Å	θ = 7.5–15°
*b* = 8.4610 (4) Å	µ = 0.30 mm^−^^1^
*c* = 19.0040 (11) Å	*T* = 291 K
*V* = 1275.89 (12) Å^3^	Block, colorless
*Z* = 4	0.30 × 0.30 × 0.20 mm

### Data collection

**Table:**

Bruker APEXII CCD diffractometer	3001 independent reflections
Radiation source: fine-focus sealed tube	2550 reflections with *I* > 2σ(*I*)
graphite	*R*_int_ = 0.033
ω scans	θ_max_ = 28.4°, θ_min_ = 3.2°
Absorption correction: multi-scan (*SADABS*; Bruker, 2001)	*h* = −10→10
*T*_min_ = 0.566, *T*_max_ = 0.716	*k* = −10→10
12886 measured reflections	*l* = −25→25

### Refinement

**Table:**

Refinement on *F*^2^	Hydrogen site location: inferred from neighbouring sites
Least-squares matrix: full	H-atom parameters constrained
*R*[*F*^2^ > 2σ(*F*^2^)] = 0.035	*w* = 1/[σ^2^(*F*_o_^2^) + (0.028*P*)^2^ + 0.4*P*] where *P* = (*F*_o_^2^ + 2*F*_c_^2^)/3
*wR*(*F*^2^) = 0.085	(Δ/σ)_max_ < 0.001
*S* = 1.01	Δρ_max_ = 0.33 e Å^−^^3^
3001 reflections	Δρ_min_ = −0.18 e Å^−^^3^
165 parameters	Extinction correction: *SHELXL97* (Sheldrick, 2008), Fc^*^=kFc[1+0.001xFc^2^λ^3^/sin(2θ)]^-1/4^
0 restraints	Extinction coefficient: 0.077 (3)
Primary atom site location: structure-invariant direct methods	Absolute structure: Flack (1983), 1255 Friedel pairs
Secondary atom site location: difference Fourier map	Flack parameter: 0.03 (7)

### Special details

**Table:**

Geometry. All e.s.d.'s (except the e.s.d. in the dihedral angle between two l.s. planes) are estimated using the full covariance matrix. The cell e.s.d.'s are taken into account individually in the estimation of e.s.d.'s in distances, angles and torsion angles; correlations between e.s.d.'s in cell parameters are only used when they are defined by crystal symmetry. An approximate (isotropic) treatment of cell e.s.d.'s is used for estimating e.s.d.'s involving l.s. planes.
Refinement. Refinement of *F*^2^ against ALL reflections. The weighted *R*-factor *wR* and goodness of fit *S* are based on *F*^2^, conventional *R*-factors *R* are based on *F*, with *F* set to zero for negative *F*^2^. The threshold expression of *F*^2^ > σ(*F*^2^) is used only for calculating *R*-factors(gt) *etc*. and is not relevant to the choice of reflections for refinement. *R*-factors based on *F*^2^ are statistically about twice as large as those based on *F*, and *R*- factors based on ALL data will be even larger.

### Fractional atomic coordinates and isotropic or equivalent isotropic displacement parameters (Å^2^)

**Table:**

	*x*	*y*	*z*	*U*_iso_*/*U*_eq_	
O1	1.51486 (16)	0.40256 (17)	0.67832 (8)	0.0512 (3)	
Cl1	1.23456 (9)	0.09826 (6)	0.64207 (3)	0.06647 (19)	
F1	0.6313 (2)	0.88072 (19)	0.64912 (8)	0.0801 (4)	
N1	0.96049 (18)	0.62572 (16)	0.64509 (8)	0.0378 (3)	
N2	1.2433 (2)	0.46187 (17)	0.70180 (8)	0.0437 (4)	
C1	0.8356 (2)	0.8253 (2)	0.56706 (9)	0.0415 (4)	
C2	0.7317 (3)	0.9332 (2)	0.59859 (10)	0.0492 (5)	
C3	0.7224 (3)	1.0901 (3)	0.58058 (12)	0.0622 (6)	
H3	0.6497	1.1582	0.6041	0.075*	
C4	0.8215 (3)	1.1433 (3)	0.52783 (12)	0.0623 (6)	
H4	0.8177	1.2491	0.5146	0.075*	
C5	0.9276 (3)	1.0405 (3)	0.49380 (12)	0.0588 (6)	
H5	0.9960	1.0766	0.4574	0.071*	
C6	0.9328 (3)	0.8832 (3)	0.51363 (10)	0.0524 (5)	
H6	1.0047	0.8148	0.4898	0.063*	
C7	0.8427 (2)	0.6546 (2)	0.58860 (10)	0.0459 (4)	
H7A	0.7312	0.6211	0.6034	0.055*	
H7B	0.8746	0.5912	0.5482	0.055*	
C8	1.1333 (2)	0.6613 (2)	0.62433 (10)	0.0424 (4)	
H8A	1.1409	0.7723	0.6118	0.051*	
H8B	1.1613	0.6000	0.5828	0.051*	
C9	1.2590 (2)	0.6266 (2)	0.68052 (10)	0.0459 (4)	
H9A	1.3719	0.6464	0.6631	0.055*	
H9B	1.2395	0.6951	0.7207	0.055*	
C10	1.0718 (2)	0.4225 (3)	0.72213 (11)	0.0500 (5)	
H10A	1.0413	0.4821	0.7638	0.060*	
H10B	1.0655	0.3109	0.7336	0.060*	
C11	0.9506 (2)	0.4583 (2)	0.66487 (11)	0.0460 (5)	
H11A	0.9758	0.3931	0.6242	0.055*	
H11B	0.8372	0.4337	0.6804	0.055*	
C12	1.3729 (2)	0.3613 (2)	0.69620 (9)	0.0388 (4)	
C13	1.3417 (3)	0.1882 (2)	0.71208 (10)	0.0476 (5)	
H13A	1.2753	0.1787	0.7547	0.057*	
H13B	1.4484	0.1351	0.7197	0.057*	

### Atomic displacement parameters (Å^2^)

**Table:**

	*U*^11^	*U*^22^	*U*^33^	*U*^12^	*U*^13^	*U*^23^
O1	0.0326 (7)	0.0537 (8)	0.0671 (9)	0.0000 (6)	−0.0001 (6)	0.0034 (7)
Cl1	0.0760 (4)	0.0506 (3)	0.0729 (3)	−0.0109 (3)	−0.0059 (3)	−0.0099 (3)
F1	0.0805 (10)	0.0910 (10)	0.0688 (8)	0.0264 (8)	0.0272 (8)	0.0187 (8)
N1	0.0326 (7)	0.0339 (7)	0.0471 (8)	0.0024 (6)	−0.0027 (6)	0.0003 (6)
N2	0.0343 (8)	0.0416 (7)	0.0552 (9)	0.0042 (7)	0.0010 (8)	0.0090 (6)
C1	0.0373 (9)	0.0480 (10)	0.0393 (9)	0.0030 (8)	−0.0091 (7)	−0.0007 (8)
C2	0.0465 (11)	0.0587 (12)	0.0423 (9)	0.0063 (10)	−0.0002 (9)	0.0045 (8)
C3	0.0751 (15)	0.0544 (11)	0.0573 (12)	0.0190 (12)	0.0006 (12)	−0.0014 (10)
C4	0.0801 (17)	0.0479 (12)	0.0590 (13)	−0.0008 (11)	−0.0167 (11)	0.0099 (10)
C5	0.0596 (14)	0.0677 (14)	0.0492 (11)	−0.0068 (11)	−0.0017 (10)	0.0128 (10)
C6	0.0502 (11)	0.0624 (13)	0.0447 (10)	0.0049 (10)	−0.0006 (9)	−0.0006 (10)
C7	0.0400 (10)	0.0464 (10)	0.0512 (10)	−0.0017 (8)	−0.0089 (9)	−0.0047 (9)
C8	0.0363 (9)	0.0337 (8)	0.0570 (11)	−0.0023 (7)	−0.0006 (8)	0.0053 (8)
C9	0.0370 (9)	0.0362 (9)	0.0645 (11)	−0.0006 (8)	−0.0076 (9)	0.0016 (8)
C10	0.0393 (11)	0.0497 (11)	0.0610 (12)	0.0068 (8)	0.0094 (9)	0.0158 (10)
C11	0.0328 (9)	0.0397 (9)	0.0656 (13)	−0.0015 (8)	0.0056 (9)	0.0061 (9)
C12	0.0364 (9)	0.0433 (9)	0.0366 (8)	0.0023 (8)	−0.0050 (7)	−0.0010 (7)
C13	0.0479 (11)	0.0442 (11)	0.0506 (11)	0.0067 (9)	−0.0049 (9)	0.0071 (9)

### Geometric parameters (Å, °)

**Table:**

O1—C12	1.228 (2)	C5—H5	0.9300
Cl1—C13	1.753 (2)	C6—H6	0.9300
F1—C2	1.324 (2)	C7—H7A	0.9700
N1—C7	1.444 (2)	C7—H7B	0.9700
N1—C8	1.459 (2)	C8—C9	1.490 (3)
N1—C11	1.467 (2)	C8—H8A	0.9700
N2—C12	1.339 (2)	C8—H8B	0.9700
N2—C10	1.454 (2)	C9—H9A	0.9700
N2—C9	1.457 (2)	C9—H9B	0.9700
C1—C6	1.366 (3)	C10—C11	1.483 (3)
C1—C2	1.369 (3)	C10—H10A	0.9700
C1—C7	1.502 (3)	C10—H10B	0.9700
C2—C3	1.373 (3)	C11—H11A	0.9700
C3—C4	1.351 (3)	C11—H11B	0.9700
C3—H3	0.9300	C12—C13	1.515 (3)
C4—C5	1.373 (3)	C13—H13A	0.9700
C4—H4	0.9300	C13—H13B	0.9700
C5—C6	1.384 (3)		
			
C7—N1—C8	111.87 (15)	C9—C8—H8A	108.9
C7—N1—C11	108.62 (14)	N1—C8—H8B	108.9
C8—N1—C11	108.59 (13)	C9—C8—H8B	108.9
C12—N2—C10	126.45 (15)	H8A—C8—H8B	107.7
C12—N2—C9	121.38 (16)	N2—C9—C8	109.27 (15)
C10—N2—C9	111.92 (15)	N2—C9—H9A	109.8
C6—C1—C2	115.21 (18)	C8—C9—H9A	109.8
C6—C1—C7	121.77 (18)	N2—C9—H9B	109.8
C2—C1—C7	123.02 (18)	C8—C9—H9B	109.8
F1—C2—C1	117.11 (18)	H9A—C9—H9B	108.3
F1—C2—C3	118.22 (19)	N2—C10—C11	111.40 (16)
C1—C2—C3	124.7 (2)	N2—C10—H10A	109.3
C4—C3—C2	118.4 (2)	C11—C10—H10A	109.3
C4—C3—H3	120.8	N2—C10—H10B	109.3
C2—C3—H3	120.8	C11—C10—H10B	109.3
C3—C4—C5	119.7 (2)	H10A—C10—H10B	108.0
C3—C4—H4	120.2	N1—C11—C10	110.55 (16)
C5—C4—H4	120.2	N1—C11—H11A	109.5
C4—C5—C6	120.0 (2)	C10—C11—H11A	109.5
C4—C5—H5	120.0	N1—C11—H11B	109.5
C6—C5—H5	120.0	C10—C11—H11B	109.5
C1—C6—C5	122.1 (2)	H11A—C11—H11B	108.1
C1—C6—H6	119.0	O1—C12—N2	123.07 (18)
C5—C6—H6	119.0	O1—C12—C13	118.68 (17)
N1—C7—C1	112.92 (15)	N2—C12—C13	118.24 (17)
N1—C7—H7A	109.0	C12—C13—Cl1	110.34 (13)
C1—C7—H7A	109.0	C12—C13—H13A	109.6
N1—C7—H7B	109.0	Cl1—C13—H13A	109.6
C1—C7—H7B	109.0	C12—C13—H13B	109.6
H7A—C7—H7B	107.8	Cl1—C13—H13B	109.6
N1—C8—C9	113.25 (16)	H13A—C13—H13B	108.1
N1—C8—H8A	108.9		
			
C6—C1—C2—F1	177.86 (18)	C11—N1—C8—C9	−57.8 (2)
C7—C1—C2—F1	−1.6 (3)	C12—N2—C9—C8	120.59 (19)
C6—C1—C2—C3	−0.9 (3)	C10—N2—C9—C8	−54.0 (2)
C7—C1—C2—C3	179.7 (2)	N1—C8—C9—N2	56.1 (2)
F1—C2—C3—C4	−178.3 (2)	C12—N2—C10—C11	−118.1 (2)
C1—C2—C3—C4	0.4 (3)	C9—N2—C10—C11	56.2 (2)
C2—C3—C4—C5	0.1 (3)	C7—N1—C11—C10	179.10 (16)
C3—C4—C5—C6	0.0 (3)	C8—N1—C11—C10	57.2 (2)
C2—C1—C6—C5	0.9 (3)	N2—C10—C11—N1	−57.6 (2)
C7—C1—C6—C5	−179.60 (19)	C10—N2—C12—O1	179.59 (18)
C4—C5—C6—C1	−0.5 (3)	C9—N2—C12—O1	5.8 (3)
C8—N1—C7—C1	−63.4 (2)	C10—N2—C12—C13	0.2 (3)
C11—N1—C7—C1	176.72 (16)	C9—N2—C12—C13	−173.64 (16)
C6—C1—C7—N1	93.1 (2)	O1—C12—C13—Cl1	−103.48 (18)
C2—C1—C7—N1	−87.4 (2)	N2—C12—C13—Cl1	75.97 (19)
C7—N1—C8—C9	−177.68 (15)		
